# Supplementary material for: MarpolBase Expression: A Web-Based, Comprehensive Platform for Visualization and Analysis of Transcriptomes in the Liverwort Marchantia polymorpha
Source: Plant Cell Physiol. 2022 Sep 9;63(11):1745–55. doi: 10.1093/pcp/pcac129 (PMC9680858; doi:10.1093/pcp/pcac129)
Supplement: pcac129_Supp [file pcac129_supp.zip › pcp-2022-e-00165-File007.docx]

**Supplementary Materials for "MarpolBase Expression: A Web-based, Comprehensive Platform for Visualization and Analysis of Transcriptomes in the Liverwort *Marchantia polymorpha"***

*Supplementary Text*

*OrthoPhyloViewer*

Users can visualize an orthologous group (orthogroup) of genes and their phylogenetic tree (Supplementary Fig. 1A, B). Five plant species are covered, i.e., *Arabidopsis thaliana*, *Oryza sativa*, *Physcomitrium patens*, *Marchantia polymorpha*, and *Klebsormidium nitens*. This link is available in the table view in the co-expression analysis.

*DataSource*

Users can search manually curated condition information and SRA samples used in MBEX. These data can be downloaded in CSV format. The raw count, TPM and correlation matrix are also available for download from the DataSource page.

*Co-Expression Viewer*

Users can visualize expression correlations between two genes (Supplementary Fig. 1D). Using the Co-Expression Viewer tool, users can examine whether two genes of interest are transcriptionally correlated or not under most or certain conditions. Furthermore, users can view the conditions under which the two selected gene pairs are co-expressed. By placing the pointer on the dot, users can see which condition the dot is in. This tool informs users about which conditions are outliers, and under which specific conditions, excluding outliers, the genes are co-expressed.

*Step-by-step instructions to reproduce the Case Study*

DEG Analysis
(“Analysis Tools” -> “Differential Expression”, or https://marchantia.info/mbex/diffexp)

1. Select and compare the following pair of conditions to generate a volcano plot and lists of DEGs
   - *Australian Melbourne thallus 21 day female mpc1hdz-2 non-axenic Romani et al., 2020* and
     *Australian Melbourne thallus 21 day female non-axenic Romani et al., 2020*
2. Download lists of both UpRegulated and DownRegulated gene IDs separately for subsequent analysis from “Download Gene List”
3. Repeat steps 1-2 above for the following pairs
   - *Tak-1 thallus 5 day male Mperf13GOF Kanazawa et al., 2020* and
     *Tak-1 thallus 5 day male Kanazawa et al., 2020*
   - *Tak-1 thallus 5 day male Mperf13ge Kanazawa et al., 2020* and
     *Tak-1 thallus 5 day male Kanazawa et al., 2020*

Set Relation Analysis for DEG analysis
(“Analysis Tools” -> “Set Relation”, or https://marchantia.info/mbex/setrel)

1. Upload all of the six files that were downloaded in the previous step
2. Click “Submit” to obtain DEGs co-expressed under the conditions selected above

Functional Network Tools for analyzing terpene biosynthesis (https://marchantia.info/mbex/functionalnetwork)

1. Set parameters under “Config” as follows
   1. Rank Type: MR (Mutual Rank)
   2. Rank Cutoff: 2000
2. Set gene names MpERF13 (Mp6g08690), MpC1HDZ (Mp3g02320), MpABCG1 (Mp8g13070), MpSYP12B (Mp4g20670)
3. Set filter word as terpene

Rank Table Analysis to explore genes co-expressed with oil body markers (https://marchantia.info/mbex/ranktable)

1. Set parameters under “Config” as follows
   1. Rank Type: MR (Mutual Rank)
   2. Rank Cutoff: 1000
2. Set gene name MpERF13 and “Submit”
3. “Download Table as CSV” to save the result as a CSV file for the subsequent analysis.
4. Repeat steps 2-3 for MpC1HDZ, MpABCG1, MpSYP12B

Set Relation Analysis for genes co-expressed with oil body markers (https://marchantia.info/mbex/setrel)

1. Upload the 4 CSV files downloaded in the previous step and “Submit”
2. Check at least three sets of genes co-expressed
3. “Download Selected Gene Information” to save the result CSV file for the subsequent analysis

Functional Enrichment Analysis (https://marchantia.info/mbex/enrichment)

1. Upload the file downloaded in the previous step and “Submit”
2. Set Annotation Type as GO for GO Enrichment
3. Enter gene names of interest and “Set Gene Names”

*Supplementary Figures*

**
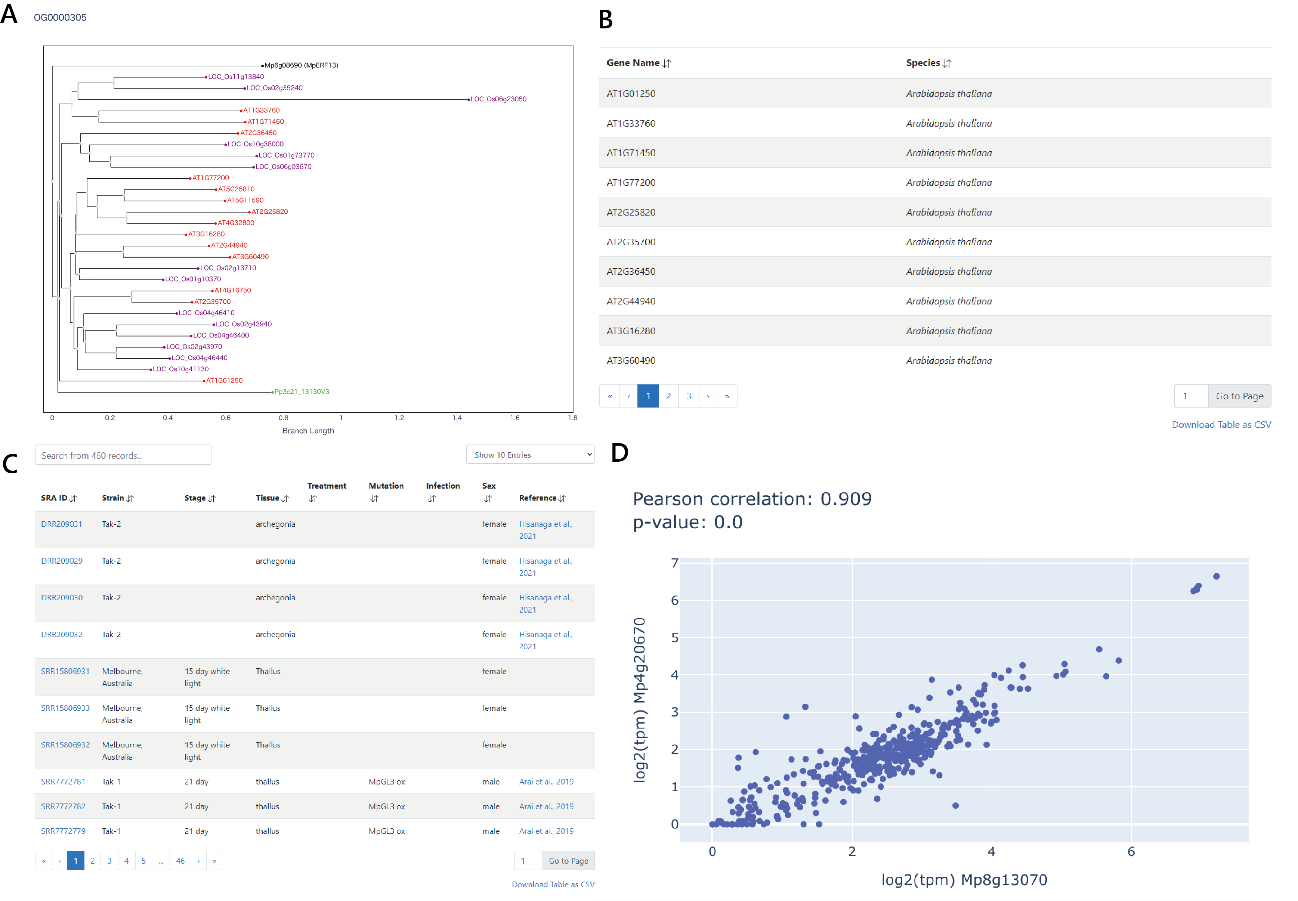
**

**Supplementary Fig. S1** **Extended tools in MBEX.**

(**A**) “OrthoPhyloViewer” shows a phylogenetic tree of the orthogroup for a given gene. (**B**) A list of genes in the orthogroup. (**C**) “Data Source” lists all the SRA samples used in MBEX for download in the CSV format. (**D**) “Co-Expression Viewer” shows expression correlations between two genes in all samples used in MBEX.


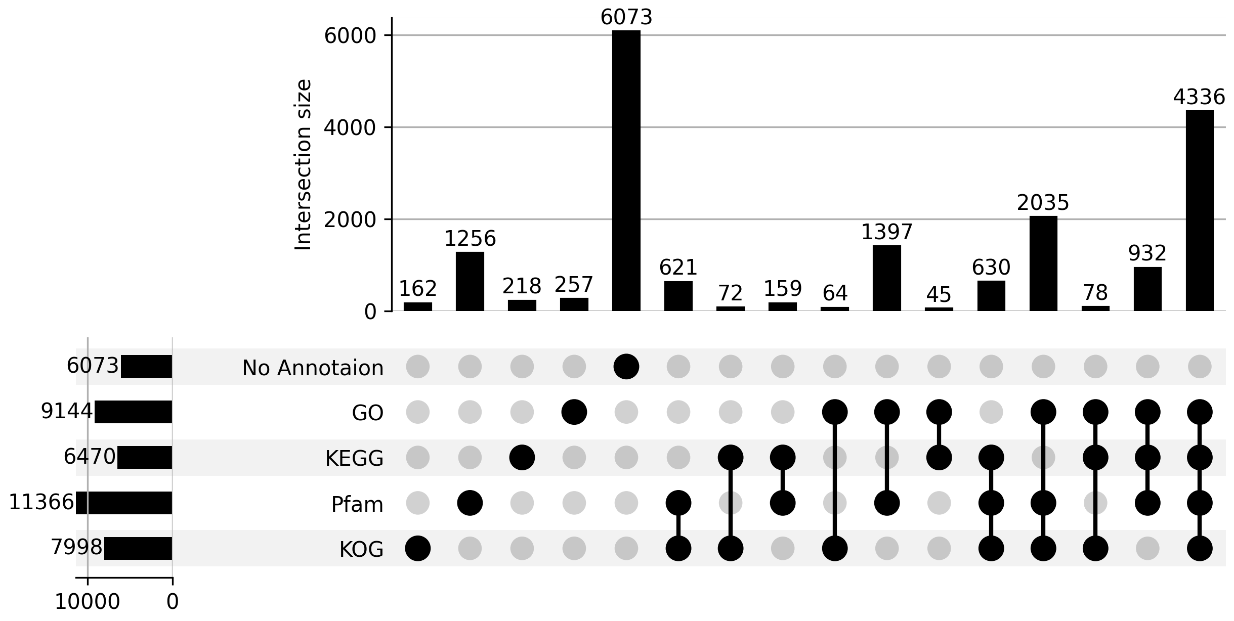


**Supplementary Fig. S2** **The annotation coverage of the *M. polymorpha* genes.**


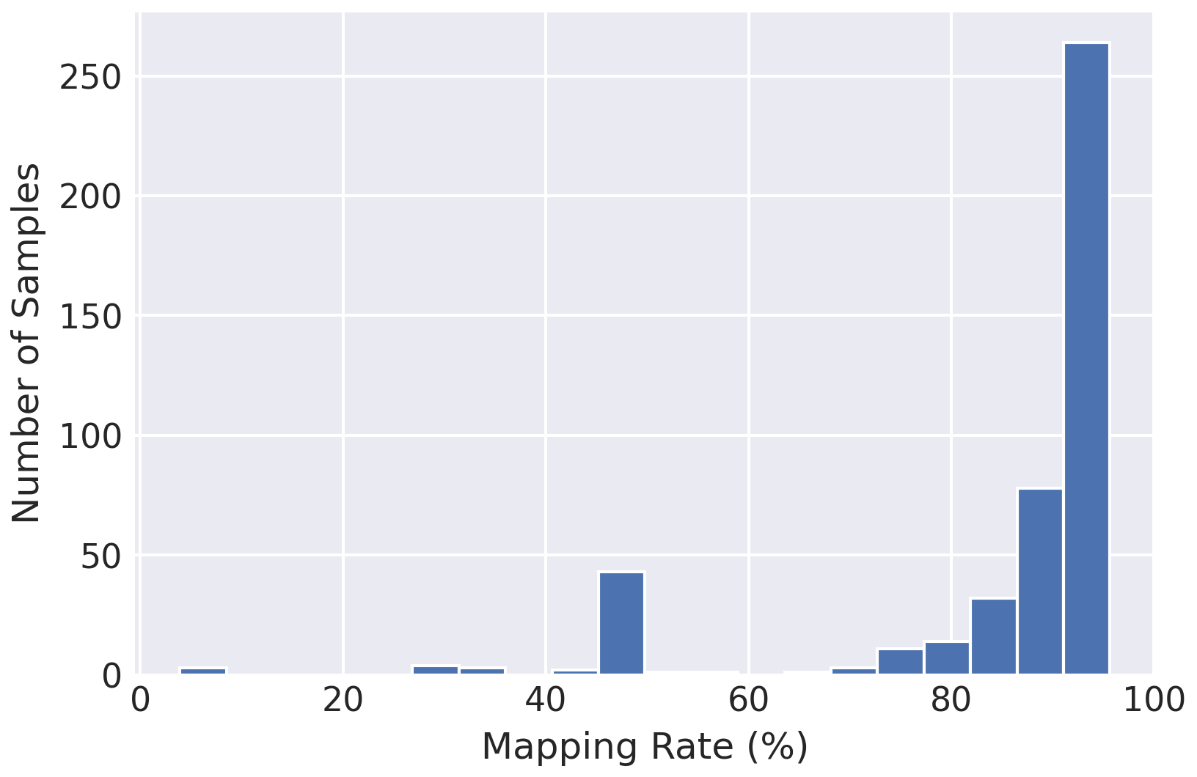
 **Supplementary Fig. S3** **Distribution of the mapping rates for the RNA-seq data used in this study.**


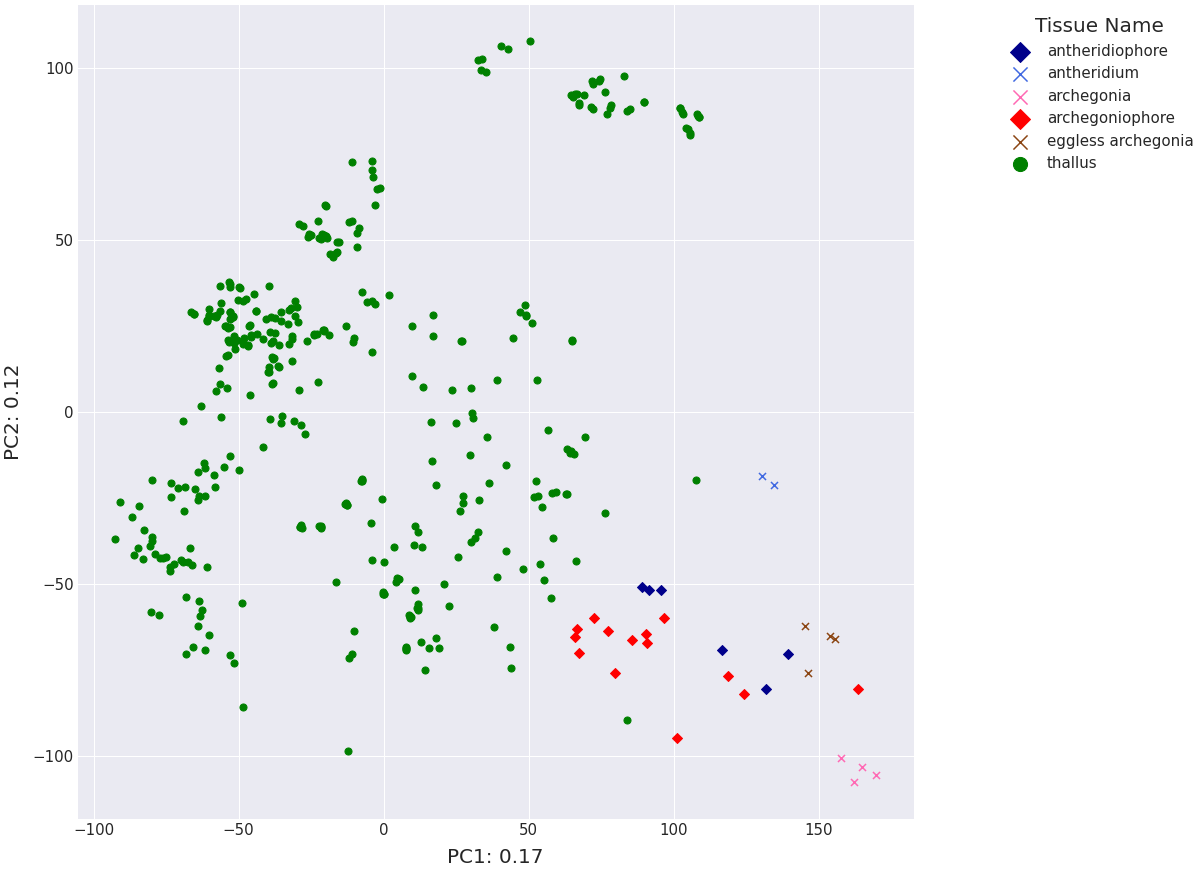


**Supplementary Fig. S4** **PCA analysis of thallus, gametangiophore, and gametangia.**

*Supplementary Table*

**Supplementary Table S1** **Sample number of each tissue.**

| Tissue Name | Sample Number |
| --- | --- |
| antheridiophore | 6 |
| antheridium | 2 |
| archegonia | 4 |
| archegoniophore | 14 |
| eggless archegonia | 4 |
| gametophytic apical cell | 3 |
| gemma | 20 |
| gemma cup | 4 |
| midrib | 3 |
| mixed | 1 |
| sperm cell | 3 |
| sporelings | 13 |
| spores | 3 |
| sporophyte | 4 |
| thallus | 376 |

*Supplementary Data*

**Supplementary Data** Mapping rates of the samples used in this study (in a separate CSV file).
